# Is There *One* Key Step in the Metastatic Cascade?

**DOI:** 10.3390/cancers13153693

**Published:** 2021-07-22

**Authors:** Antoine M. Dujon, Jean-Pascal Capp, Joel S. Brown, Pascal Pujol, Robert A. Gatenby, Beata Ujvari, Catherine Alix-Panabières, Frédéric Thomas

**Affiliations:** 1 CREEC/CANECEV, MIVEGEC (CREES), University of Montpellier, CNRS, IRD, 34172 Montpellier, France; p-pujol@chu-montpellier.fr (P.P.); frederic.thomas2@ird.fr (F.T.); 2 Centre for Integrative Ecology, School of Life and Environmental Sciences, Deakin University, Waurn Ponds, VIC 3216, Australia; beata.ujvari@deakin.edu.au; 3 Toulouse Biotechnology Institute, INSA, CNRS, INRAE, University of Toulouse, 31555 Toulouse, France; capp@insa-toulouse.fr; 4 Department of Integrated Mathematical Oncology, Moffitt Cancer Center, Tampa, FL 33601, USA; Joel.Brown@moffitt.org (J.S.B.); Robert.Gatenby@moffitt.org (R.A.G.); 5 Oncogenetic Department, University Medical Centre of Montpellier, 34000 Montpellier, France; 6 School of Natural Sciences, University of Tasmania, Hobart, TAS 7000, Australia; 7 Laboratory of Rare Human Circulating Cells (LCCRH), University Medical Centre of Montpellier, 34000 Montpellier, France

**Keywords:** metastatic process, Drake equation, circulating tumor cells, metastasis

## Abstract

**Simple Summary:**

To successfully metastasize, cancer cells must complete a sequence of obligatory steps called the metastatic cascade. To model the metastatic cascade, we used the framework of the Drake equation, initially created to describe the emergence of intelligent life in the Milky way, using a similar logic of a sequence of obligatory steps. Then within this framework, we used simulations on breast cancer to investigate the contribution of each step to the metastatic cascade. We show that the half-life of circulating tumor cells is one of the most important parameters in the cascade, suggesting that therapies reducing the survival of those cells in the vascular system could significantly reduce the risk of metastasis.

**Abstract:**

The majority of cancer-related deaths are the result of metastases (i.e., dissemination and establishment of tumor cells at distant sites from the origin), which develop through a multi-step process classically termed the metastatic cascade. The respective contributions of each step to the metastatic process are well described but are also currently not completely understood. Is there, for example, a critical phase that disproportionately affects the probability of the development of metastases in individual patients? Here, we address this question using a modified Drake equation, initially formulated by the astrophysicist Frank Drake to estimate the probability of the emergence of intelligent civilizations in the Milky Way. Using simulations based on realistic parameter values obtained from the literature for breast cancer, we examine, under the linear progression hypothesis, the contribution of each component of the metastatic cascade. Simulations demonstrate that the most critical parameter governing the formation of clinical metastases is the survival duration of circulating tumor cells (CTCs).

## 1. Introduction

Metastases, the spread of malignant cells from a primary tumor to distant organs, remain the leading cause of cancer morbidity and mortality [1]. Identifying effective ways to prevent metastases would significantly benefit cancer patients but there is currently no consensus on optimal strategies [2,3,4].

Decades of investigations have demonstrated that the metastatic process is a multi-step process (termed the “metastatic cascade”) that includes a series of distinct and necessary events. The cascade is usually divided into: (1) local invasion of malignant cells into surrounding tissue; (2) intravasation, i.e., entrance into the circulatory and lymphatic systems; (3) survival within the circulatory system; (4) extravasation, i.e., exit of circulating tumor cells (CTCs) from the bloodstream into adjacent normal tissue; and, (5) survival and then proliferation leading to colonization and macro-metastases [5,6,7]. Both clinical observations and pre-clinical experiments have demonstrated that successful completion of all steps in the metastatic cascade is very rare, and that the vast majority of cells leaving a tumor fail to colonize distant organs [8,9,10].

The precise reason(s) for this inefficiency remains unclear, but it seems likely that successful completion of the metastatic cascade requires selection forces to undergo multiple events (e.g., [11]) with cancer cell adaptations that are not previously experienced in primary cancer, each necessitating genetic and/or epigenetic alterations as well as co-option of stromal and endothelial cells [7,12] from tissues different from the primary site. However, because the number of cancer cells in the primary tumor is large and because of inherent genotypic and phenotypic heterogeneity, there is a finite probability that some cells will survive the events of the cascade and form colonies in distant organs [8].

While all steps in the previously described chain-of-events are undoubtedly important for the metastatic cascade, it remains unclear if certain steps represent a disproportionately larger barrier to success. If so, treatment-related variations, even small, in one of these critical steps could have a major impact on the probability of metastases [13]. Initial experimental studies [14] have concluded the rate-limiting steps in the metastatic cascade are the transition of solitary cells to micrometastases and then to macroscopic tumors at the metastatic site. Yet this approach has investigated only the last steps of the process because the cancer cells were directly injected intraportally to target the mouse liver. Multiscale computational models were later employed to capture the whole invasion–metastasis cascade and provide insights into the mechanisms underpinning the metastatic process at the cellular scale [7,15]. However, these models rapidly grew in complexity, requiring significant amounts of computational power, with 10’s to 100’s of numerical parameters that can be difficult to interpret individually.

Here, we therefore addressed this question using a parsimonious and original modelling approach, which is relatively easy to implement and adapted from the Drake equation proposed by astrophysicists in the 1960’s to evaluate the probability of another complex phenomenon occurring, the emergence of intelligent civilizations in the Milky Way [16]. Using numerical and sensitivity analyses calibrated from data published on invasive breast cancers, we explored and quantified the relative importance of the different steps in the metastatic cascade and showed that the most essential parameter to the success of metastasis, by far, is the life-expectancy of CTCs during their journey in the bloodstream.

## 2. Material and Methods

### 2.1. The Drake Equation

The Drake equation is a probabilistic argument formulated by Frank Drake in 1961 [16] and is used as a thought experiment to estimate the number N  of active, communicative, extra-terrestrial civilizations in the Milky Way galaxy. At its core, the equation describes the emergence of an intelligent civilization as a series of sequential steps. Those steps include the formation of a solar system with a planet that can support life, the emergence of intelligence on this planet, and the ability for an intelligent species to develop a technology that releases detectable signs of their existence for a long enough period of time that they may be detected [16,17]. The equation is as follows:

(1) $$\;N={\;R}_{*}\cdot fp\cdot ne\cdot f1\cdot fi\cdot fc\cdot L$$

With $R_{*}$ the average rate of star formation in our galaxy (in stars per year), fp the fraction of those stars that have planets, ne the average number of planets that can potentially support life per star that has planets, f1 the fraction of planets that could support life that actually develop life at some point, fi the fraction of planets with life that actually go on to develop intelligent life, fc the fraction of civilizations that develop a technology that release detectable signs of their existence into space, and L the length of time for which such civilizations release detectable signals into space (in years). While no intelligent extraterrestrial civilization has been discovered so far, because of its relative simplicity, the Drake equation has been very successful in generating discussions since its creation. Indeed, the logic of a sequence of obligatory steps required for a phenomenon to emerge (and the associated equations) which is famously illustrated by the Drake equation, can be applied to a range of topics, including cancer cell metastases.

### 2.2. The Metastatic Drake Equation

To successfully metastasize, cancer cells shedding from a primary tumor site must complete a sequence of obligatory steps, called the metastatic cascade, which comprises: detachment from the primary tumor, intravasation into the vascular system, survival while in transit through the circulation, extravasation, and survival and proliferation in the target tissue [18]. Because of the sequential nature of the metastatic cascade, we can reformulate the Drake equation, which we now term the “metastatic Drake equation” to estimate the number of cells and provide quantification for each step of the cascade. A very similar approach of modifying the Drake equation was recently used to estimate the number of mammal and bivalve species on Earth in which transmissible cancer emerged [19]. The metastatic Drake equation is defined as follows:

(2) Ne=M·Nc·Pi·Pc·Pe·L

**Figure 1:**
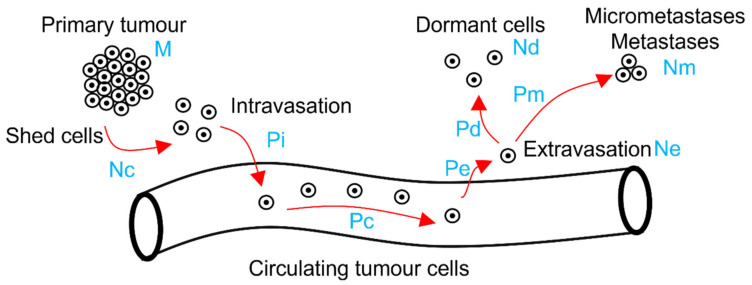
Illustration of the different steps of the metastatic cascade. Each step is represented by a red arrow. The blue text represents the associated parameters of the metastatic Drake equation.

First, we estimated the number of cells Ne surviving the travel to the target organ in the vascular system and that successfully extravasate as (Figure 1):

With M the mass of the primary tumor in grams, Nc the number of cells shed by the tumor in the number of cells per gram per day, Pi the probability that a detached cell intravasates into the vascular system, Pc the probability that a CTC survives in the vascular system and reaches the target organ, Pe the probability that a CTC that reached the target organ extravasates, and L is the age of the tumor in days (i.e., time since the formation of the tumor). The model assumes a linear progression of cancer in which cells acquire full malignancy within the tumor environment before disseminating to distant sites [20]. The model also assumes that each step of the metastatic cascade is independent of the others (i.e., the model uses conditional probabilities). It also assumes that the values of all the parameters (except M and L) remain constant during the lifetime of the tumor. In addition, this modified equation aggregates the complex phenomenon of the metastatic cascade under a relatively small number of parameters that can be experimentally measured (and for which we can find values in the literature). While in this study we used point estimates from the literature (using a similar approach as [17]), we can assume that each parameter of the equation could be drawn from a given probability distribution (for example a Poisson distribution for M and Nc or a binomial distribution for Pi, Pc and Pe in Equation (2) and Pd and Pm in Equations (3) and (4) below).

After calculating Ne, the number Nd of cells that successfully extravasate and will become dormant cells can then be calculated as (Figure 1):

(3) Nd=Ne·Pd

In which Pd is the probability that an extravasated cell becomes dormant.

(4) Nm=Ne·Pm

In which Pm is the probability that an extravasated cell survives extravasation and forms a metastasis without undergoing dormancy.

Alternatively, more complex models can be considered in which a cell successfully extravasates and survives to develop into a micrometastasis but then become dormant, regress or simply die.

Based on these equations, the number of dormant cells and metastases is directly dependent on the number of CTCs Ne that successfully extravasate in the target organ. It is, therefore, of key importance to estimate the effect of these parameters on the predictions returned by the equation.

### 2.3. Parameters Estimation from the Literature

In order to populate the metastatic Drake equation with numerical values and estimate the number Ne of cells that successfully extravasate in the target organ, we screened the literature to identify cancer types in which experiments quantifying one or more steps of the metastatic cascade were conducted. After a preliminary screening, we selected breast cancer as a study case for which we found numerical values for most steps of the metastatic cascade and because breast cancer is the most common cancer, in women, worldwide [21]. In the metastatic Drake equation defined above, the product Nc·Pi which quantifies the number of cells that are shed from the primary tumor site and intravasate in the vascular system to become CTCs was estimated to be 3.18 × 10^6^ or 4.05 × 10^6^ cells per gram of tumor per day [22]. The probability Pc that a CTC survives in the circulating system was calculated based on the half-life of 1 and 2.5 h, estimated by [23], and using the following equation:

(5) $$Pc=0.5^{\frac{t}{\lambda }}$$

With *t*, the time elapsed since a cell extravasated, and λ the half-life of CTCs (in hours). The probability Pe that a CTC that reached the target organ extravasated was estimated to be 0.236 [24], 0.384 [25] and 0.56 or 0.22 [26] based on a range of in-vivo and in-vitro extravasation experiments.

### 2.4. Numerical Simulations

We considered two-time scales, first, a period of 24 h to investigate the interaction between the time taken by CTCs to reach the target organ and the parameters of the metastatic Drake equation; and second, a six-month time scale to investigate how tumor growth interacts with those same parameters.

#### 2.4.1. Twenty-Four-Hour Time Scale

In the following simulations, we tested for all possible combinations of parameters obtained from the literature for each step of the equation. This allowed us to investigate which steps of the metastatic cascade had the most influence on the predicted number of extravasated cells and to account for the variability between estimates. This approach of testing for all available combinations was previously used to estimate the number of intelligent civilizations present in the Milky way based on the various estimates provided in the literature for the original Drake equation [17]. All 24-hour long simulations were performed assuming that the tumor remained at a constant size of 1 g.

Because the half-life of CTCs is only a maximum of few hours, they are rapidly eliminated from the vascular system and after 24 h almost all cells are expected to be dead [23]. Therefore, the time elapsed between the intravasation in the vascular system and the extravasation in the target organ is predicted to be critical to the success of the metastatic process. We then investigated the effect of both CTC half-life (ranging from 15 min to 6 h) and the time that it took CTCs to reach the target organ once they entered the vascular system (0 to 200 min) on the number of cells that extravasate after 24 h. The median number of extravasated cells as a function of the time CTCs take to reach the target organ was calculated for each half-life value. Similarly, the proportion of CTCs dying in the vascular system was computed for each simulation.

Then, to investigate the effect of a potential therapy designed to reduce the half-life of CTCs, the median number of extravasated cells and the proportion of CTCs dying in the vascular system were compared in simulations for which the half-life of CTCs was reduced by a potential treatment (by either 15 min or 30 min) to simulations in which treatment was absent.

#### 2.4.2. Six-Month Time Scale

To investigate how the tumor growth rate interacted with the other metastatic Drake equation parameters, we repeated the simulations above but allowed the tumor to grow over time. We first considered a case in which the number of cells that shed from the primary tumor site and intravasate in the vascular system (the product Nc·Pi) is proportional to the tumor volume. In the second case, we considered that the number of cells a tumor sheds is scaling with the mass of the volume (e.g., M^b^) with an exponent b representing a very small fraction of the tumor volume (here 0.001). This scenario aimed to simulate a situation where metastases are descendants of stem-like cancer cells with stable population sizes due to asymmetric division. Finally, in the third case, we considered that the number of cells a tumor sheds scales with its surface, assuming a spherical tumor shape. An initial tumor weight of 1 g shedding 3.18 × 10^6^ or 4.05 × 10^6^ cells per day was assumed to have a volume of 75.7 mm^3^, a radius of 2.6 mm and a surface of 86.5 mm^2^ (based on [27]). This equates to a number of 36,763 or 46,821 cells that intravasate in the vascular system per mm^2^ per day (according to [22]).

For the three scenarios, the CTCs were considered to have a half-life of 1 or 2.4 h [23] and all the probabilities of extravasation Pe in the target organs were the same as the ones used for the 24 h temporal scale. In addition, for the three scenarios, we defined a tumor volume increase of either 1.003% per day for a triple negative breast cancer, which is considered to be a fast growing breast cancer type or of 0.175% per day for a Luminal A breast cancer which is considered to be a slower growing cancer type [28]. The scenarios were computed for a duration of six months, starting with a tumor of 1 g (180 days, with a simulated tumor volume, for the triple-negative breast cancer, increasing from 75.7 mm^3^ to 456.3 mm^3^ with a radius increasing from 2.6 mm to 4.8 mm; and, for the luminal A cancer, a tumor volume increasing from 75.7 mm^3^ to 103.7 mm^3^ with a radius increasing from 2.6 to 2.9 mm). For each day of the simulations, we computed the median number of cells that successfully extravasated in the target organ based on all possible parameter combinations for a given simulation.

All simulations and statistical analyses in this publication were performed using R software, version 4.0.2.

## 3. Results

### 3.1. 24-Hour Time Scale

#### 3.1.1. Simulating the Number of Extravasated Cells as Function of Half-Life and Time to Reach the Target Organ

**Figure 2:**
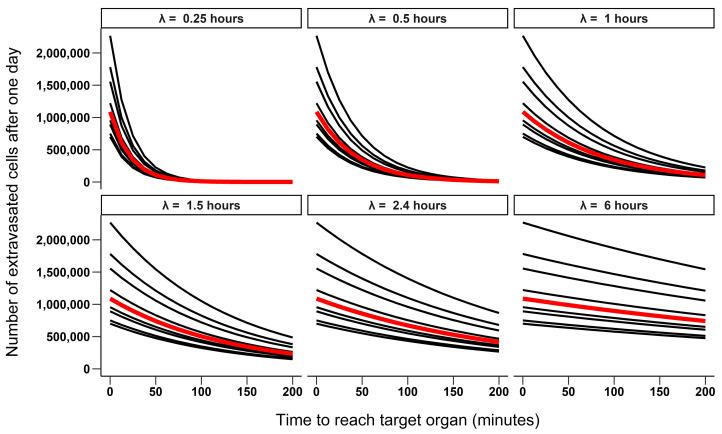
The number of extravasated cells predicted by calculating all avalaible parameter combinations obtained from the literature as function of the time CTCs take to reach the target organ and their half-life (ranging from 0.25 to 6 h). Each solid line corresponds to one combination of parameters, and the bold red line to the median number of extravasated cells over time for each half-life value. Values for Pe (the probability of a CTC extravasating in the target organ) were set to 0.236, 0.384, 0.56 or 0.22. A tumor mass M of 1 g was assumed, and the number of cells shed from a tumor this size was assumed as 3.18 × 10^6^ or 4.05 × 10^6^ cells per gram of tumor per day.

**Figure 3:**
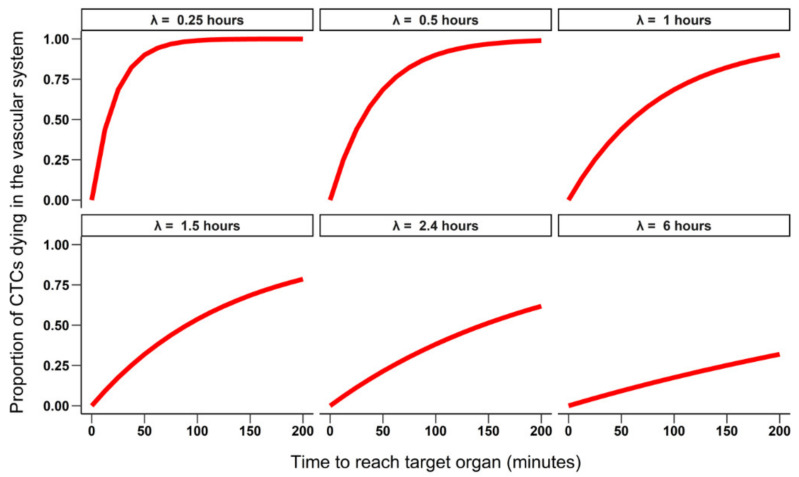
Proportion of CTCs dying in the vascular system as a function of their half-life (ranging from λ = 0.25 to 6 h) and the time required to reach the target organ (0 to 200 min). The values for M, Pe and the number of shed cells are the same as in Figure 2.

We observed two patterns in the simulated scenarios. First, the half-life of CTCs was the parameter having the largest effect on the number of extravasated cells when their lifetime was short (half-life < 1.5 h, Figure 1), with most cells dying in the vascular system (Figure 2). Second, as the duration of the half-life increased (e.g., >2.4 h), leaving more time for the cells to reach the target organ, its importance in the equation decreased and at least half of the cells survived and reached the target organ. The importance of the other steps of the metastatic cascade in the equation, therefore, increased as evidenced by the increasing variability in the predicted number of extravasated cells between simulations (Figure 2 and Figure 3). The simulations also indicated that the half-life of CTCs had little effect on their mortality if they very quickly reached the target organ (i.e., within a few minutes).

#### 3.1.2. Simulating the Effect of a Treatment on the Survival and Extravasation of CTCs

**Figure 4:**
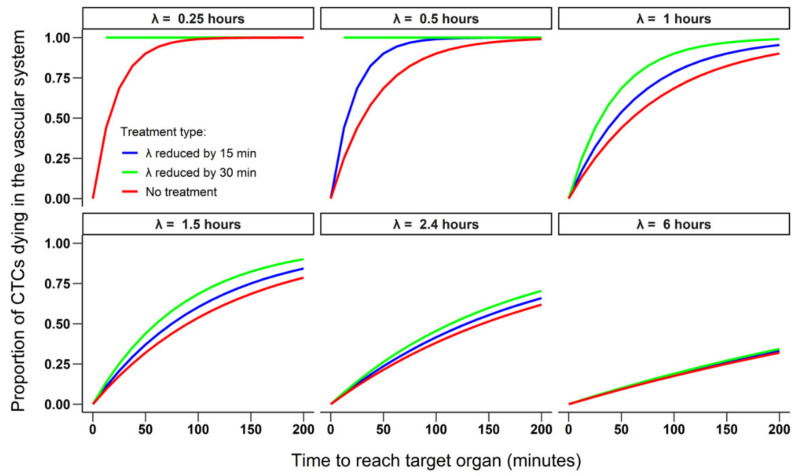
The effect of a hypothetical treatment on the proportion of CTCs dying in the vascular system as function of their half-life (initially ranging from 0.25 to 6 h) and the time required to reach the target organ. The red lines correspond to mortality calculated from simulations in which no treatment was applied, the blue lines from simulation in which the half-life was reduced by 15 min and the green lines from simulation in which the half-life was reduced by 30 min. The values for M, Pe and the number of shed cells are the same as in Figure 2. Note that in the simulation with a half-life of 0.25 h, the green line (half-life reduced by 30 min) overlaps the blue line (half-life reduced by 15 min).

Simulations investigating the effect of potential therapies showed that even small reductions in the half-life of CTCs (e.g., by 15 to 30 min) can have an important effect on the metastatic cascade and prevent 100,000 s to 1,000,000 s of cells per day from reaching the target organ and extravasating (Figure 3 and Figure 4). This is particularly true within the range of half-life values reported in the literature for breast cancer (1 to 2.4 h [23]). For CTCs with a relatively long half-life (which have yet to be observed in vivo), the reduction in survivability would need to be more substantial (a reduction in half-life by at least 4 h would obtain the same effect as for the shorter-lived CTCs). Using a treatment reducing the half-life of CTCs in the vascular system could, therefore, be an efficient strategy to decrease the number of cells that extravasate in the target organ (Figure 3 and Figure 4).

### 3.2. Six-Month Time Scale

**Figure 5:**
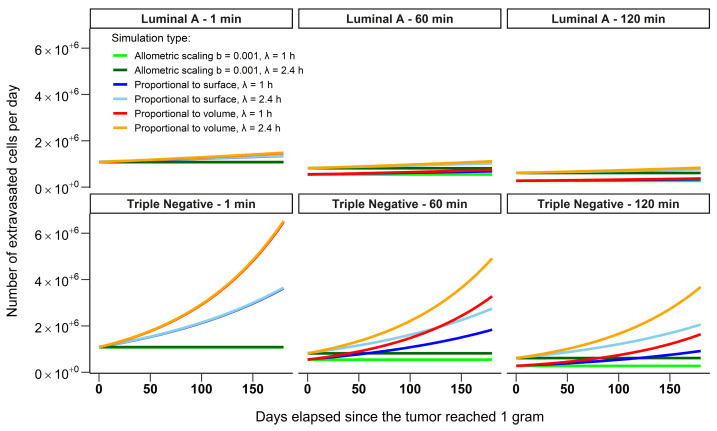
The number of extravasated cells per day for two different breast cancer types: triple-negative cancer which is fast growing and luminal A which is slow-growing. CTCs with half-lives of 1 and 2.4 h and a time for those cells to reach the target organ of 1, 60 or 120 min were considered in the simulation. The solid lines represent the median number of extravasated cells per day for a given simulation. Luminal A tumors grew by 0.175% per day while triple-negative cancer tumors grew by 1.003% per day. Note that the two lines for the simulations scaling to a small fraction of the tumor mass M (here M^b^ with b = 0.001) (in green and dark green) are overlapping closely on the plots. The values for M, Pe and the number of shed cells are the same as in Figure 2.

For the two cancer types (fast-growing triple-negative and slow-growing luminal A), a median of 1.5 times more cells will extravasate per day if they have a half-life of 2.4 h and take 60 min to reach the target organ compared to those with a half-life of 1 h. This ratio increases to 2.25 if the time to reach the target organ is increased to 120 min, with a higher proportion of cells dying in the vascular system. If CTCs reach the target organ almost immediately (i.e., in 1 min, Figure 5) almost all parameters of the equations have little effect on the median number of extravasated cells per day. The predicted number of extravasated cells is then primarily driven by whether the number of cells is calculated as proportional to the volume or the surface area of the tumor with a relatively low number of cells shed if proportional to a very small fraction of the tumor volume. Six months after it reached a mass of 1 g a triple-negative breast cancer will shed a median of 2.7–4.4 times more cells that will extravasate per day (based on the tumor surface and volume respectively) compared to a luminal A tumor because of the difference in size between the tumor types (451.8 mm^3^ compared to 103.7 mm^3^, Figure 5). This ratio range remains constant at any time between the two cancer types regardless of the half-life of CTCs or the time that CTCs take to reach the target organ. Those results suggest that reducing the half-life of CTCs early in the development of the tumor has the potential to prevent a very large number of CTCs to extravasate in the target organ, particularly for fast-growing tumors.

## 4. Discussion

As proposed by the Drake equation, the likelihood of life on any given planet is tiny, but the likelihood of life on other planets becomes larger once multiplied by the vast number of planets in our galaxy [16]. For similar reasons, the logic behind the Drake equation applies to the steps of the metastatic cascade; the progression from a primary tumor to the metastatic disease represents a sequence of relatively independent and highly improbable events, at least for a given cancer cell [29]. Our extension of the Drake equation to metastases provides insights into the rate-limiting steps and identifies bottlenecks where changes in certain parameters have outsized effects on the probability of occurrence and time to form clinically relevant metastases. For breast cancer, and by considering the linear progression model rather than the parallel one (see [30]), two key elements of the metastatic cascade include the half-life of CTCs and the likelihood of a small group of cells successfully establishing within the tissue of extravasation.

In breast cancer, the likelihood of any given cell from the primary tumor forming a metastasis is infinitesimal. However, if left untreated, metastases to the liver, bone, and/or lungs become almost certain. As such, it becomes a numbers game. While the rate of cell intravasation into the circulatory system increases with tumor size, the actual allometry of this relationship remains unknown. We imagine a relationship of the form *N*_C_ = *aM^b^* where −1 < *b* < 0 scales this release by total tumor mass. Under such a formulation, the number of tumor cells released per day per gram of primary tumor, Nc, declines with tumor mass, *M*, but the overall number of released cells, M·Nc, increases with tumor mass.

Metastases do not seem to be propagule-limited in that enormous numbers of cancer cells will become CTCs, even when the number of cells shed is a very small fraction of the tumor [29]. The circulatory system acts as a sink habitat because cancer cells cannot proliferate and maintain a positive population in the absence of migration from the source habitat (the primary tumor). Thus, the rate of change in the number of CTCs is dictated by the per cell immigration rate from the primary tumor, the death rate in the circulatory system, and the rate of extravasation into any tissue of the patient. If the number of CTCs equilibrates much faster than the growth of the primary tumor volume, then the number of CTCs will equal M·Nc·Pi divided by the sum of the death rate and extravasation rate. This becomes a useful relationship for estimating and validating some of the terms in our modified Drake Equation. For instance, CTCs can range between 1–10 per mL [31,32]. About 10% of a person’s body mass is comprised of blood which amounts to 4500–5700 mL per person. At ca. 5000 mL, the number of CTCs would be 5000–10,000. For metastatic cancers such as breast cancer, a CTC count of 5 or more per 7.5 mL of blood is associated with lower progression-free survival [33,34]. If one knows the number of CTCs, then the rates at which cancer cells enter the bloodstream, die in the blood, or leave the blood are no longer completely independent variables.

There are a number of assumptions associated with applying our version of the Drake equation [35]. The successfully metastasizing cell may be just a subset of the cancer cell subtypes found in the primary tumor. In addition to tumor size, greater tumor heterogeneity, the presence of highly glycolytic phenotypes, and the presence of immunosuppressive phenotypes are associated with more aggressive cancers and higher rates of metastases. There is also debate over whether disseminated tumor cells (DTCs) occur early or later in the development of the primary tumor [36], as in breast cancer there is evidence that DTCs occur relatively early in the history of the tumor [37,38]. If early, then these cells may have remained dormant or persisted as small undetectable populations for lengthy periods of time suggesting that outgrowth is a key limiting step in addition to colonizing a distant organ. Alternatively, if later, then most CTCs and DTCs perish and key steps remain survival in the blood and survival as a single cell or small propagule of cells in the following colonization. Traits such as the endothelial–mesenchymal transition (EMT) [36], glycolysis, immuno-evasion, motility, and the ability to survive the shear forces of the circulatory system all may create a weighted lottery for which cancer cells actually form successful metastases. Some of these traits may facilitate multiple steps of the metastatic cascade or in some cases facilitate one step while inhibiting another. For our modelling purposes, the number of tumor cells can be triaged for those that have substantially more favorable values in the Drake-like equation. Alternatively, parallel equations could be made for the relevant subsets of cancer cell types.

Other considerations of how cells metastasize include the mechanical phenotype, the role of CTCs as solitary cells or as small clumps of cells, and polyaneuploid cancer cells (PACC). For example, Kumar et al. [39] discussed the role of actin mechanics and mechanisms for degrading and constructing extracellular matrices in inhibiting or facilitating a cancer cell’s chances throughout the metastatic cascade. Invadopodia facilitate intravasation, cytoskeleton plasticity may facilitate surviving shear forces in the bloodstream, and pseudopodia may permit extravasation via diapedesis (the ability to pass through cell–cell junctions). CTCs as clumps of two or more cells have the disadvantage perhaps of lodging in capillary beds and smaller blood vessels more easily than a single cell; but clumps may extravasate more easily and form successful colonies by overcoming Allee effects and more quickly engineering a more favorable extracellular matrix at the new site [40,41,42]. Finally, multinucleated PACCs may represent a highly resistant and motile life history state of a cancer cell line. These have been proposed as the source of metastases [43,44]. They generally represent only a small fraction of the cancer cells within a tumor [45]. As the roles of these subsets of cells and phenotypes become known, the rates and probabilities associated with the metastatic cascade can be adjusted accordingly. By modelling all steps of the metastatic cascade in the form of a Drake equation, one can quantitatively evaluate the efficacy of therapies aimed at preventing metastases. In recognizing the inefficiency of the metastatic process, therapies have been proposed or even implemented to target particular steps. Additional approaches have been suggested in order to alter tumor microenvironments, reduce heterogeneity, and alter the evolutionary trajectory of cancer cells in a manner that reduces the rate of intravasation and thus the number of CTCs. One example is the addition of bicarbonate therapy to reduce tumor pH. This disfavors the more motile and glycolytic cancer cell types and favors those that are less motile and differentiated [46,47]. Rates of intravasation increase with transforming growth factor-beta signaling, possibly by accelerating EMT or activating epidermal growth factor receptors. Furthermore, invadopodia emerge from signaling involving Phosphoinositide 3-Kinase, Neural Wiskott–Aldrich Syndrome Protein, Ras homolog family member A, and Wiskott–Aldrich Syndrome Protein [48], and targeting these pathways has been shown to reduce intravasation and metastases in a mouse model [49].

In our model, the most effective therapies would be those that reduce the half-life of CTCs and that prevent extravasation and colonization. At present, targeting colonization has attracted the most attention [49]. A promising set of therapies involves immunotherapies that can be tailored to target CTCs or increase immune surveillance in tissues that are likely recipients of CTCs shed from the primary tumor [50]. Because cytotoxic therapies in the blood will likely have adverse effects and because the numbers of CTCs are quite small, immune activation therapies may be the only viable approach to kill CTCs before they extravasate [51].

Neoadjuvant therapies and adjuvant therapies prior to and after either surgical resection or radiation of the primary tumor already serve to destroy micrometastases or DTCs. Because establishing metastases, though undetectable, can still involve millions of cells, evolutionarily informed therapies may help with increasing the number and switching between drugs as has been proposed for breast cancer [52]. Furthermore, because cancer cells must take on some of the attributes typical of the recipient tissue, it might be effective to use drugs typically associated with cancers of that tissue. For instance, DTCs or micrometastases in the liver might be susceptible to drugs not typically used for breast cancer, but rather typical of primary liver cancers [53].

Quantifying and applying our model has value in evaluating the efficacy of combination therapies that target different steps of the metastatic cascade. Because the steps are multiplicative in terms of rates and survival probabilities, the independent action of several drugs impacting several steps will have multiplicative benefits. Thus, the action of one therapy increases the value of another. Further, the action of each drug need not be decisive by itself but become so in combination. Our sensitivity analyses permit joint consideration of changing two or more parameters at a time.

Our model assumes independent sequences of rates and probabilities, an assumption that is not necessarily realistic for all subsets of cancer cells. This concern can be alleviated by either restricting the model to the critical cell types or by running parallel equations for the different conditions. An alternative approach that could complement ours would involve working backward from successful metastases or micrometastases to identify the crucial subsets of cancer cell types or conditions favoring the metastatic cascade. This can be done through phylogenetic reconstruction that traces the cancer cells within the metastasis to their ancestral cell types in the primary tumor [20].

Numerical estimates for the parameters of the metastatic Drake equation are only available in the literature for a few cancer types (with breast cancer being the most complete). Due to this lack of data, it is currently difficult to apply the equation to simulate parallel cancer progression models, an avenue that remains to be explored. It is then of interest to obtain estimates for a broader range of cancers, either by conducting experiments or by using more complex but also much more computationally intensive models that would specifically focus on a specific step of the metastatic cascade. The metastatic Drake equation could be then applied to those cancer types to investigate the effect of a range of potential therapeutic scenarios. Whether predicting forward as we do here or backward, modelling all of the sequential steps of the metastatic cascade as a multiplicative chain allows one to better understand which steps have the highest leverage in predicting or manipulating the likelihood of metastases.

## 5. Conclusions

From a preventive point of view, our study proposes promising directions. Indeed, it suggests that, administrating to people in the second part of life, when most cancers appear, a systematic medication aimed at reducing the life expectancy of CTCs should be highly protective against the eventuality of metastatic cancers. To potentially reduce the side-effects of regular preventive therapy, future research should be performed which specifically targets the life expectancy of metastasis-initiator CTCs.

## Data Availability

Data is contained within the article.

## References

[1] Dillekås H., Rogers M.S., Straume O. (2019). Are 90% of deaths from cancer caused by metastases?. Cancer Med..

[2] Bohl C.R., Harihar S., Denning W.L., Sharma R., Welch D.R. (2014). Metastasis suppressors in breast cancers: Mechanistic insights and clinical potential. J. Mol. Med..

[3] Khan I., Steeg P.S. (2018). Metastasis suppressors: Functional pathways. Lab. Investig..

[4] Stoletov K., Beatty P.H., Lewis J.D. (2020). Novel therapeutic targets for cancer metastasis. Expert Rev. Anticancer Ther..

[5] Lambert A.W., Pattabiraman D.R., Weinberg R.A. (2017). Emerging biological principles of metastasis. Cell.

[6] Eslami S.Z., Majidzadeh A.K., Halvaei S., Babapirali F., Esmaeili R. (2020). Microbiome and breast cancer: New role for an ancient population. Front. Oncol..

[7] Franssen L.C., Lorenzi T., Burgess A.E.F., Chaplain M.A.J. (2019). A mathematical framework for modelling the metastatic spread of cancer. Bull. Math. Biol..

[8] Ganesh K., Massagué J. (2021). Targeting metastatic cancer. Nat. Med..

[9] Chambers A.F., Groom A.C., MacDonald I.C. (2002). Dissemination and growth of cancer cells in metastatic sites. Nat. Rev. Cancer.

[10] Mehlen P., Puisieux A. (2006). Metastasis: A question of life or death. Nat. Rev. Cancer.

[11] Arnal A., Ujvari B., Crespi B., Gatenby R.A., Tissot T., Vittecoq M., Ewald P.W., Casali A., Ducasse H., Jacqueline C. (2015). Evolutionary perspective of cancer: Myth, metaphors, and reality. Evol. Appl..

[12] Valastyan S., Weinberg R.A. (2011). Tumor metastasis: Molecular insights and evolving paradigms. Cell.

[13] Dujon A.M., Aktipis A., Alix-Panabières C., Amend S.R., Boddy A.M., Brown J.S., Capp J., DeGregori J., Ewald P., Gatenby R. (2021). Identifying key questions in the ecology and evolution of cancer. Evol. Appl..

[14] Luzzi K.J., MacDonald I.C., Schmidt E.E., Kerkvliet N., Morris V.L., Chambers A.F., Groom A.C. (1998). Multistep nature of metastatic inefficiency: Dormancy of solitary cells after successful extravasation and limited survival of early micrometastases. Am. J. Pathol..

[15] Warner H.V., Sivakumar N., Peirce S.M., Lazzara M.J. (2019). Multiscale computational models of cancer. Curr. Opin. Biomed. Eng..

[16] Drake F.D. Discussion at space science board, national academy of sciences. Proceedings of the Conference on Extraterrestrial Intelligent Life.

[17] Sandberg A., Drexler E., Ord T. (2018). Dissolving the Fermi Paradox. arXiv.

[18] Hunter K.W., Crawford N.P., Alsarraj J. (2008). Mechanisms of metastasis. Breast Cancer Res..

[19] Dujon A.M., Bramwell G., Roche B., Thomas F., Ujvari B. (2020). Transmissible cancers in mammals and bivalves: How many examples are there?. BioEssays.

[20] Naxerova K., Jain R.K. (2015). Using tumour phylogenetics to identify the roots of metastasis in humans. Nat. Rev. Clin. Oncol..

[21] Bray F., Ferlay J., Soerjomataram I., Siegel R.L., Torre L.A., Jemal A. (2018). Global cancer statistics 2018: GLOBOCAN estimates of incidence and mortality worldwide for 36 cancers in 185 countries. CA Cancer J. Clin..

[22] Butler T.P., Gullino P.M. (1975). Quantitation of cell shedding into efferent blood of mammary adenocarcinoma. Cancer Res..

[23] Meng S., Tripathy D., Frenkel E.P., Shete S., Naftalis E.Z., Huth J.F., Beitsch P.D., Leitch M., Hoover S., Euhus D. (2004). Circulating tumor cells in patients with breast cancer dormancy. Clin. Cancer Res..

[24] Chen M.C., Whisler J.A., Jeon J.S., Kamm R.D. (2013). Mechanisms of tumor cell extravasation in an in vitro microvascular network platform. Integr. Biol..

[25] Jeon J.S., Bersini S., Gilardi M., Dubini G., Charest J.L., Moretti M., Kamm R.D. (2015). Human 3D vascularized organotypic microfluidic assays to study breast cancer cell extravasation. Proc. Natl. Acad. Sci. USA.

[26] Martin M.D., Kremers G.-J., Short K.W., Rocheleau J.V., Xu L., Piston D.W., Matrisian L.M., Gorden D.L. (2010). Rapid extravasation and establishment of breast cancer micrometastases in the liver microenvironment. Mol. Cancer Res..

[27] Esteva-Font C., Jin B.J., Verkman A.S. (2014). Aquaporin-1 gene deletion reduces breast tumor growth and lung metastasis in tumor-producing MMTV-PyVT mice. FASEB J..

[28] Lee S.H., Kim Y.S., Han W., Ryu H.S., Chang J.M., Cho N., Moon W.K. (2016). Tumor growth rate of invasive breast cancers during wait times for surgery assessed by ultrasonography. Medicine.

[29] Lloyd M.C., Gatenby R.A., Brown J.S. (2017). Ecology of the Metastatic Process. Ecology and Evolution of Cancer.

[30] Nguyen D.X., Bos P.D., Massagué J. (2009). Metastasis: From dissemination to organ-specific colonization. Nat. Rev. Cancer.

[31] Yu M., Stott S., Toner M., Maheswaran S., Haber D.A. (2011). Circulating tumor cells: Approaches to isolation and characterization. J. Cell Biol..

[32] Alix-Panabières C., Pantel K. (2021). Liquid biopsy: From discovery to clinical application. Cancer Discov..

[33] Rack B., Schindlbeck C., Jückstock J., Andergassen U., Hepp P., Zwingers T., Friedl T.W.P., Lorenz R., Tesch H., Fasching P.A. (2014). Circulating tumor cells predict survival in early average-to-high risk breast cancer patients. JNCI J. Natl. Cancer Inst..

[34] Pantel K., Alix-Panabières C. (2019). Liquid biopsy and minimal residual disease—Latest advances and implications for cure. Nat. Rev. Clin. Oncol..

[35] Pantel K., Speicher M.R. (2016). The biology of circulating tumor cells. Oncogene.

[36] Pantel K., Alix-Panabières C. (2017). Tumour microenvironment: Informing on minimal residual disease in solid tumours. Nat. Rev. Clin. Oncol..

[37] Hanin L., Bunimovich-Mendrazitsky S. (2014). Reconstruction of the natural history of metastatic cancer and assessment of the effects of surgery: Gompertzian growth of the primary tumor. Math. Biosci..

[38] Hanin L., Korosteleva O. (2010). Does extirpation of the primary breast tumor give boost to growth of metastases? Evidence revealed by mathematical modeling. Math. Biosci..

[39] Kumar S., Weaver V.M. (2009). Mechanics, malignancy, and metastasis: The force journey of a tumor cell. Cancer Metastasis Rev..

[40] Aceto N., Bardia A., Miyamoto D.T., Donaldson M.C., Wittner B.S., Spencer J.A., Yu M., Pely A., Engstrom A., Zhu H. (2014). Circulating tumor cell clusters are oligoclonal precursors of breast cancer metastasis. Cell.

[41] Castro-Giner F., Aceto N. (2020). Tracking cancer progression: From circulating tumor cells to metastasis. Genome Med..

[42] Celià-Terrassa T., Kang Y. (2018). Metastatic niche functions and therapeutic opportunities. Nat. Cell Biol..

[43] Pienta K.J., Hammarlund E.U., Brown J.S., Amend S.R., Axelrod R.M. (2021). Cancer recurrence and lethality are enabled by enhanced survival and reversible cell cycle arrest of polyaneuploid cells. Proc. Natl. Acad. Sci. USA.

[44] Pienta K.J., Hammarlund E.U., Axelrod R., Brown J.S., Amend S.R. (2020). Poly-aneuploid cancer cells promote evolvability, generating lethal cancer. Evol. Appl..

[45] Mallin M.M., Pienta K.J., Amend S.R. (2020). Cancer cell foraging to explain bone-specific metastatic progression. Bone.

[46] Ibrahim-Hashim A., Robertson-Tessi M., Enriquez-Navas P.M., Damaghi M., Balagurunathan Y., Wojtkowiak J.W., Russell S., Yoonseok K., Lloyd M.C., Bui M.M. (2017). Defining cancer subpopulations by adaptive strategies rather than molecular properties provides novel insights into intratumoral evolution. Cancer Res..

[47] Robey I.F., Baggett B.K., Kirkpatrick N.D., Roe D.J., Dosescu J., Sloane B.F., Hashim A.I., Morse D.L., Raghunand N., Gatenby R.A. (2009). Bicarbonate increases tumor pH and inhibits spontaneous metastases. Cancer Res..

[48] Chiang S.P.H., Cabrera R.M., Segall J.E. (2016). Tumor cell intravasation. Am. J. Physiol. Cell Physiol..

[49] Abdul Pari A.A., Singhal M., Augustin H.G. (2021). Emerging paradigms in metastasis research. J. Exp. Med..

[50] Yang H., Kuo Y., Smith Z.I., Spangler J. (2021). Targeting cancer metastasis with antibody therapeutics. WIREs Nanomed. Nanobiotechnol..

[51] Jacot W., Mazel M., Mollevi C., Pouderoux S., D’Hondt V., Cayrefourcq L., Bourgier C., Boissiere-Michot F., Berrabah F., Lopez-Crapez E. (2020). Clinical correlations of programmed cell death ligand 1 status in liquid and standard biopsies in breast cancer. Clin. Chem..

[52] Artzy-Randrup Y., Epstein T., Brown J.S., Costa R.L., Czerniecki B.J., Gatenby R.A. (2021). Novel evolutionary dynamics of small populations in breast cancer adjuvant and neoadjuvant therapy. NPJ Breast Cancer.

[53] Cunningham J.J., Brown J.S., Vincent T.L., Gatenby R.A. (2015). Divergent and convergent evolution in metastases suggest treatment strategies based on specific metastatic sites. Evol. Med. Public Health.

